# The Characteristics of Youth With Missed HIV Visits in Alabama

**DOI:** 10.1093/ofid/ofae086

**Published:** 2024-02-14

**Authors:** Jiaying Hao, Dustin M Long, Heather M Relyea Ashley, Henna Budhwani, Tina Y Simpson, Samantha V Hill

**Affiliations:** Department of Biostatistics, School of Public Health, University of Alabama at Birmingham, Birmingham, Alabama, USA; Department of Pediatrics, School of Medicine, University of Alabama at Birmingham, Birmingham, Alabama, USA; Department of Biostatistics, School of Public Health, University of Alabama at Birmingham, Birmingham, Alabama, USA; Department of Pediatrics, School of Medicine, University of Alabama at Birmingham, Birmingham, Alabama, USA; College of Nursing, Florida State University, Tallahassee, Florida, USA; Department of Pediatrics, School of Medicine, Tulane University, New Orleans, Louisiana, USA; Department of Pediatrics, School of Medicine, University of Alabama at Birmingham, Birmingham, Alabama, USA

**Keywords:** HIV, missed visits, retention in care, youth

## Abstract

Gaps in knowledge remain related to understanding missed human immunodeficiency virus (HIV) visits and youth with HIV (YWH). This study examined data from an Alabama academic HIV clinic with clients aged 16 to 24 years old and found that non virally suppressed and older YWH were associated with missed visits among YWH.

The Southern United States accounted for 51% of new human immunodeficiency virus (HIV) diagnoses in the United States in 2020 [1] secondary to its large and geographically dispersed population and social and political determinates of health that impact healthcare access and outcomes. The Alabama Health Department states new diagnoses are shifting toward younger age groups and calls for increased prevention efforts (eg, youth with HIV [YWH] reaching undetectable viral loads [VL]) among younger populations [2]. YWH have lower rates of testing, diagnosis, treatment engagement, and viral suppression (VS) compared with adults with HIV [3] and an estimated 6% of YWH have suppressed HIV VL [4]. Associations between poor retention in care and higher risk of morbidity and mortality have also been identified among YWH [5, 6]. Importantly, unique developmental, psychosocial, behavioral, and infrastructural factors affect this vulnerable age group [7].

For people with HIV (PWH), retention in care is a crucial factor for optimal clinical outcomes (eg, undetectable VL), among which missed scheduled visits is an important measure. Significant associations between missed visits with treatment failure and mortality have been identified [7–11]. Clients with missed visits are more likely to have interruptions in antiretroviral therapy, resulting in negative treatment outcomes, including lower CD4 counts, virologic failure, and coinfections with other sexually transmitted infections [9, 11–20]. Identifying clients at greater risk of missing HIV visits could prompt healthcare providers to preemptively intervene and help these clients identify strategies for improved adherence and treatment outcomes. Although studies have focused on PWH's appointment attendance and risk factors for missed visits, including some highlighting younger clients with HIV being more likely to miss visits than older counterparts, few studies have specifically examined YWH's missed appointment visits [11, 21]. This study was designed to explore characteristics of YWH who miss HIV visits.

## METHODS

This retrospective study occurred within an Alabama academic center HIV clinic that provides HIV treatment and wraparound services to mothers, infants perinatally infected, children, adolescents, young adults up to age 30 years, and adult women with HIV [22].

Eligibility criteria for YWH engaged in care at the clinic were:

1. Established appointment records within the electronic medical record (EMR).
2. Complete demographic information and laboratory records during the study period.
3. Age 16 to 24 years at their first service during the study period.

Client records from 1 March 2020 to 31 August 2021 were extracted from the EMR. Appointment status was documented as show, rescheduled, or no-show, and starting 1 March 2020 included both in-person and telehealth appointments from the coronavirus disease 2019 (COVID-19) pandemic. Client appointments were made by case managers and all clients received reminder phone calls from staff within 7 days of their appointments.

The study's primary outcome was missed visit, which was any no-show visit without prior notice or rescheduling or any visit rescheduled for more than 30 days after the original scheduled visit. Missed visits were coded as a dichotomous variable. The term “index visit” represents the first record or first visit during the study period to avoid confusion with “initial visit” because in this study, most of these first visits were not initial visits to establish care. Age (in years as a continuous variable), gender, race, HIV transmission mode, insurance type, and VL were variables extracted from the EMR. VS was defined as having less than 200 copies of HIV per milliliter of blood.

Age, gender, race, HIV transmission mode, insurance type, and VS at index visits were included in bivariate and multivariable logistic regression models to identify all associated factors associated with client missed visits. Because of small sample size, some groups were collapsed. Odds ratios (OR), 95% confidence intervals (CI), and *P* values were reported. All analyses were performed with SAS 9.4.

## RESULTS

A total of 101 clients aged 16–24 years at their index visit were found with appointment records from 1 March 2020 to 31 August 2021. Five clients were excluded because they did not have a VL at their index visit. Among 96 clients, the majority were male (69.8%), African-American (72.9%), and men who have sex with men (58.3%). This study included both in-person clinic visits and telehealth virtual visits. Among 454 visit records, only 15 were telehealth visits, with 1 missed and the other 14 attended. Fifty-one clients (53.1%) had at least 1 missed visit (Table 1).

**Table 1.** Demographic Information of Clients (N = 96)

Variable		Clients
Age	Mean (SD)	20.90 (2.40)
Gender	Female	24 (25.00%)
	Male	67 (69.79%)
	Transgender	1 (1.04%)
	Unsure	4 (4.17%)
Race	AA	70 (72.92%)
	White	17 (17.71%)
	Asian	2 (2.08%)
	More than 1 race	4 (4.17%)
	Unsure	3 (3.13%)
HIV transmission mode	Heterosexual	18 (18.75%)
	MSM	56 (58.33%)
	Perinatal	17 (17.71%)
	MSM and SUI	1 (1.04%)
	Unsure	4 (4.17%)
Insurance type	Medicaid	19 (19.79%)
	Private individual	41 (42.71%)
	Private employer	10 (10.42%)
	VA, Tricare, and other military health care	2 (2.08%)
	Other	3 (3.12%)
	No insurance	8 (8.33%)
	Unsure	13 (13.54%)
Missed visits	0	45 (46.88%)
	1	30 (31.25%)
	2–3	15 (15.63%)
	4–5	6 (6.25%)
Index viral suppression	Yes	53 (55.21%)
	No	43 (44.79%)

Characteristics are summarized from their first visit record during the study period. Data are presented as n (%). Viral suppression was defined as having less than 200 copies of HIV per milliliter of blood.

Abbreviations: AA, African-American; MSM, men who have sex with men; SUI, substance-using individuals.

With unadjusted logistic models, clients without VS at index visits had high missed visit rates compared to those virally suppressed (*P* = .04; OR = 2.44; 95% CI, 1.06–5.56). With adjusted modeling, older age, and absence of VS at index visit appeared to be significantly associated with missed visits (*P* = .02; OR = 1.33; 95% CI, 1.05–1.69; *P* = .02; OR = 3.45; 95% CI, 1.22–10.00) in multivariable models. Gender, race, HIV transmission mode, and insurance type were not significantly associated with missed visits in unadjusted or adjusted models (Table 2).

**Table 2.** Risk Factors Associated With Missed Visits Among YWH (N = 96)

Effect	OR (95% CI)	*P*	AOR (95% CI)	*P*
Age (in y)		.32		.**02**
	1.09 (0.92–1.28)		1.33 (1.05–1.69)	
Gender		.29		.68
Male versus female or other^a^	1.27 (0.82–1.97)		1.40 (0.29–6.88)	
Race		.44		.24
African-American versus White	1.70 (0.58–4.97)		1.60 (0.42–6.04)	
Other^b^ versus White	2.86 (0.53–15.47)		5.69 (0.75–43.19)	
HIV risk factor		.41		.33
Heterosexual and other versus perinatal	0.94 (0.26–3.34)		0.24 (0.04–1.58)	
MSM^c^ versus perinatal	1.55 (0.52–4.59)		0.62 (0.13–2.94)	
Insurance type		.64		.39
Medicaid versus private—individual	0.96 (0.32–2.85)		1.55 (0.37–6.46)	
Private—employer versus private—individual	0.37 (0.08–1.64)		0.37 (0.07–2.01)	
Other^d^ versus private—individual	1.36 (0.44–4.20)		2.42 (0.55–10.75)	
No insurance versus private—individual	1.44 (0.30–6.83)		0.91 (0.15–5.46)	
Viral suppression at index visit		.**04**		.**02**
No versus yes	2.44 (1.06–5.56)		3.45 (1.22–10.00)	

Bivariate and multivariable logistic regressions were conducted. Odds ratios, 95% confidence intervals, and *P* values were reported. Bold *P* value indicates significance at *P* < .05.

Abbreviations: CI, confidence interval; MSM, men who have sex with men; SUI, substance-using individuals; OR, odds ratio; YWH, youth with HIV.

^a^Female, transgender, and unsure were combined to “female or other.”

^b^Asian, more than 1 race, and unsure were combined to “other.”

^c^The client under the MSM and SUI group was assigned to MSM group in data analysis to avoid sample size issue.

^d^The clients with VA, Tricare, and other military healthcare and unsure were combined to “other.”

## DISCUSSION

YWH are a distinct subgroup of PWH secondary to their continual development cognitively, physically, and psychosocially [23]. Thus, YWH require more focused studies on their retention in care and health outcomes. Older YWH and those not virally suppressed were more likely to have missed visits. Understanding factors influencing HIV visit adherence has the potential to reduce the risk of further HIV transmission, improve long-term health outcomes in YWH, and aid in ending the pandemic by contributing to the Ending the Epidemic Plan for America goals [24].

This study's findings are similar to other studies focused on YWH where associations between antiretroviral therapy adherence and VS with missed clinic visits was found [25–27]. One study of YWH from the United States and Puerto Rico found the following YWH to be more likely to miss 2 or more visits: Black, Mixed Race, female, those with disclosed HIV status, those with unknown HIV transmission modes, and those using substances [3]. Even though this other study may have been better powered to detect these differences, the study was conducted among clinics serving major metropolitan cities whereas this study includes YWH throughout a predominately rural Alabama. Furthermore, studies suggest improved social support is associated with improved retention [3, 25]. Additionally, more youth-tailored strategies able to keep YWH engaged in care are needed.

Additionally, certain demographics were associated with missed visits; older youth were found to have greater likelihood of missed appointments [28]. Tarantino et al. also found that older age approached significance [3]. Research on PWH (older than age 18 years) found that the younger age groups were more likely to have missed visits [11, 17, 29, 30]. Early deployed, patient-centered interventions focused on when youth engage in care have the potential to be impactful in retaining YWH in care, and thus should be 1 area where future research efforts are focused. Furthermore, interventions such as Data to Care [31], which focuses on identifying and linking PWH into care and assisting these clients with overcoming barriers to viral suppression may be even more important among YWH subgroups.

The COVID-19 pandemic has significantly impacted clinic visits for a wide range of health conditions, including HIV. To minimize potential exposure to COVID-19, many healthcare providers rapidly transitioned to virtual visits as a safer alternative to in-person visits. Unfortunately, this study was not powered to compare in-person versus telehealth visit attendance with health outcomes. However, it is also important to mention that although most clients were offered telehealth visits during the height of the pandemic, many preferred in-person visits. Further exploration of the connection between appointment attendance and virtual visits is needed for healthcare providers to develop interventions to improve retention in care.

Although this is 1 of few studies focusing specifically on YWH living in a rural state, this study had some limitations. First, although inclusion of a longer time span would have been ideal, neither the clinic nor the EMR tracked visit status until March 2020, hindering the ability to include any pre-COVID data and ascertainment of how the pandemic, specifically, affected visit attendance. Furthermore, certain demographic and client characteristics were unavailable (eg, year of enrollment/diagnosis, substance abuse, psychiatric disorders), which could be potential risk factors for missed visits in YWH. It is also important to note that the age range of YWH is not standardized in literature. This study chose to use the World Health Organization definitions in which individuals 15 to 24 years of age are considered youth [32]. Additionally, this study focused on an age range where youth were able to take more responsibility for their behavior. Given the legal driving age in Alabama is 16 [33], using 16 as the lower limit of cutoff was appropriate. However, even with this cutoff, potential bias caused by parental involvement (eg, transportation, appointment reminders, medication reminders) was likely. Additionally, this study had a small sample size and was focused on YWH within a single state, limiting the generalizability of the findings.

Thus, older YWH and those without VS at the index visit may be at additional risk for missed visits. More youth-tailored interventions are needed to identify challenges to keeping YWH engaged in care and virally suppressed.

## Notes

***Acknowledgment***. We acknowledge the team at Alabama academic center HIV clinic, specifically Dayna Cooks-Heard, who have provided full support for this study.

***Authors’ Contributions***. J. H.: Statistical Conceptualization (lead), Data Curation, Investigation, Formal Analysis, Writing-Original Draft Preparation. D. L.: Conceptualization (supporting), Supervision (lead), Writing-Review and Editing. H. R. A.: Investigation (supporting), Writing-Review and Editing. H. B.: Substantive Conceptualization; Supervision (supporting), Writing-Review and Editing. T. S.: Data Ownership; Writing-Review and Editing. S. H.: Senior Author, Writing-Review and Editing.

***Patient Consent Statement***. The protocol and project materials were reviewed and approved by the local ethics committee: the institutional review board of the University of Alabama at Birmingham (IRB-981112002). The IRB waived the need to document informed consent for this retrospective study involving reviewing de-identified electronic medical record information.

***Consent for publication***. Not applicable.

***Data availability***. The datasets used and/or analyzed during the current study are available from the corresponding author on reasonable request.
